# Improving activity of minicellulosomes by integration of intra- and intermolecular synergies

**DOI:** 10.1186/1754-6834-6-126

**Published:** 2013-08-30

**Authors:** Qi Xu, Shi-You Ding, Roman Brunecky, Yannick J Bomble, Michael E Himmel, John O Baker

**Affiliations:** 1 Biosciences Center, National Renewable Energy Laboratory, Golden, Colorado 80401, USA

**Keywords:** Cellulosomes, Engineered minicellulosomes, Cellulase, Intra- and intermolecular synergies

## Abstract

**Background:**

Complete hydrolysis of cellulose to glucose requires the synergistic action of three general types of glycoside hydrolases; endoglucanases, exoglucanases, and cellobiases. Cellulases that are found in Nature vary considerably in their modular diversity and architecture. They include: non-complexed enzymes with single catalytic domains, independent single peptide chains incorporating multiple catalytic modules, and complexed, scaffolded structures, such as the cellulosome. The discovery of the latter two enzyme architectures has led to a generally held hypothesis that these systems take advantage of intramolecular and intermolecular proximity synergies, respectively, to enhance cellulose degradation. We use domain engineering to exploit both of these concepts to improve cellulase activity relative to the activity of mixtures of the separate catalytic domains.

**Results:**

We show that engineered minicellulosomes can achieve high levels of cellulose conversion on crystalline cellulose by taking advantage of three types of synergism; (1) a complementary synergy produced by interaction of endo- and exo-cellulases, (2) an intramolecular synergy of multiple catalytic modules in a single gene product (this type of synergism being introduced for the first time to minicellulosomes targeting crystalline cellulose), and (3) an intermolecular proximity synergy from the assembly of these cellulases into larger multi-molecular structures called minicellulosomes. The binary minicellulosome constructed in this study consists of an artificial multicatalytic cellulase (CBM4-Ig-GH9-X1_1_-X1_2_-GH8-Doc) and one cellulase with a single catalytic domain (a modified Cel48S with the structure CBM4-Ig-GH48-Doc), connected by a non-catalytic scaffoldin protein. The high level endo-exo synergy and intramolecular synergies within the artificial multifunctional cellulase have been combined with an additional proximity-dependent synergy produced by incorporation into a minicellulosome demonstrating high conversion of crystalline cellulose (Avicel). Our minicellulosome is the first engineered enzyme system confirmed by test to be capable of both operating at temperatures as high as 60°C and converting over 60% of crystalline cellulose to fermentable sugars.

**Conclusion:**

When compared to previously reported minicellulosomes assembled from cellulases containing only one catalytic module each, our novel minicellulosome demonstrates a method for substantial reduction in the number of peptide chains required, permitting improved heterologous expression of minicellulosomes in microbial hosts. In addition, it has been shown to be capable of substantial conversion of actual crystalline cellulose, as well as of the less-well-ordered and more easily digestible fraction of nominally crystalline cellulose.

## Background

Lignocellulose-derived sugars represent the largest reserve of fermentable sugars in Nature. However, the diversity and complexity of lignocellulose, both chemically and structurally, contribute significantly to its recalcitrance to deconstruction [1]. Cellulolytic organisms have evolved several mechanisms to overcome this natural recalcitrance in order to utilize these lignocellulose-derived sugars. To date, three dominant types of cellulase systems have been identified, each using specific sets of enzymatic synergisms [2]. The most common is the free cellulase system, which exists mainly in cellulolytic fungi and bacteria and uses mainly intermolecular endo-, and exo-cellulase synergy [3]. The next most common system is characterized by its multicatalytic independent cellulases and is typical of some cellulolytic bacteria, such as *Caldicellulosiruptor sp.*[4,5]. These multicatalytic cellulase systems utilize the synergism between nearby exo- and endo- cellulases by combining these activities into a single gene product [4]. And finally, the least common system, the cellulosome system found in some anaerobic bacteria and rumen fungi, utilizes a tethered multi-enzyme proximity-dependent synergy [6-9]. *Clostridium thermocellum* is a well-studied producer of cellulosomes, known to be extremely large and complex self-assembling arrays of as many as 91 cellulase enzymes [10,11].

Many attempts have been made to exploit and improve these natural paradigms for biomass deconstruction using artificial cellulase systems [12]. Studies have shown that some artificial multicatalytic cellulases can exhibit higher activity than their natural counterparts. It has also been demonstrated that intramolecular synergy exists in these artificial multifunctional cellulases, and that construction of artificial multicatalytic cellulases is a practical approach to improve cellulase activity [13,14]. Similarly, cellulosomes, due to their high cellulolytic activity, have inspired many studies for designing “engineered minicellulosomes,” which are smaller and simpler versions of the cellulosome, as tools for understanding the action of the more complex natural system [15-17]. Another potential use for these minicellulosomes lies in tailoring the cellulosomal organization to act optimally against specific substrates for practical applications. Specific minicellulosomes, built both *in vitro* and *in vivo,* have been used for the digestion of model crystalline cellulose (Avicel) as well as real plant cell walls [16,18-21].

Minicellulosomes with defined subunit compositions have previously been constructed in vitro [15-17,22,23]. A number of these constructs have displayed readily-measurable activity against cellulosic substrates, with some notable examples demonstrating significant proximity synergy, in that the multi-subunit constructs show greater activities than do the equivalent simple mixtures of the individual constituent enzymes [22,23]. Interpretation of these results in terms of general saccharification of “crystalline cellulose”, however, suffers from limitations imposed by the relatively low extents of conversion achieved. In terms of action against Avicel, for example, the observed percent-conversion by the most active of the minicellulosomes is under 6% [23]. Given that Avicel may contain as much as 40% amorphous cellulose [24], it is not certain that these constructs, as assayed, have been shown to be truly capable of degrading crystalline cellulose. Other recent efforts have described the creation of organisms that employ designed minicellulosomes, and as a result are capable of utilizing various cellulosic materials and growing and even producing ethanol from them [16,18-21,25,26].

These earlier minicellulosomes were built using cellulosomal cellulases that have a single catalytic module in each individual enzyme, and this limitation in the number and variety of catalytic modules in minicellulosomes may play a role in limiting the activities measured. Thus, to enhance the overall activity of these defined minicellulosomes, a variety of cellulosomal cellulases (i.e., more diversity in activity) should be incorporated. However, the co-expression of multiple recombinant genes in microbes required to assemble complex minicellulosomes with many cellulosomal enzymes is problematic. It is therefore critical to find approaches to building stable, highly active minicellulosomes with fewer individual component enzymes. To this end, we propose to improve minicellulosome design by utilizing a reduced number of individual multicatalytic cellulases.

## Results

### Construction of a multifunctional cellulase with high intramolecular synergy

**Figure 1:**
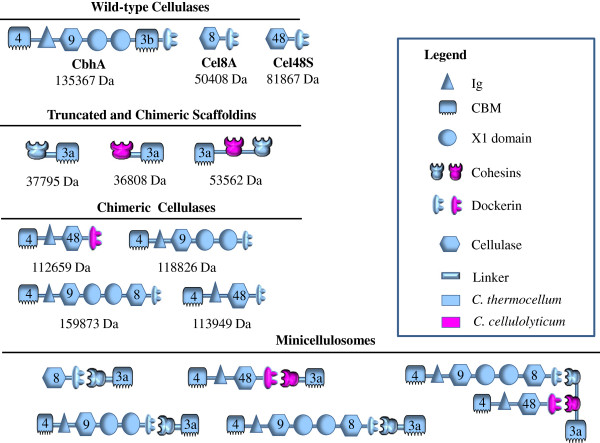
Pictorial key to the components of the cellulases, scaffoldins and minicellulosomes prepared in this study.

**Figure 2:**
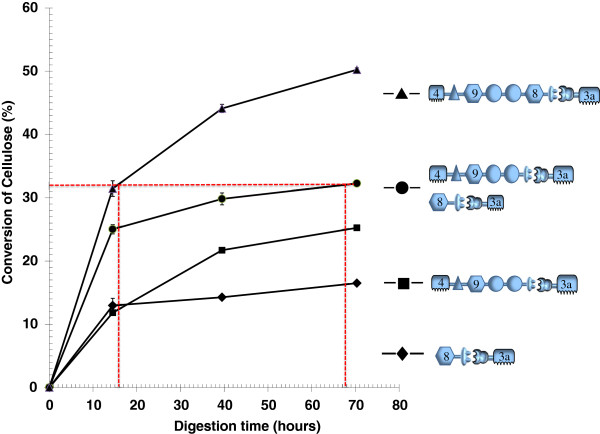
**Avicelase activities of the artificial bi-catalytic cellulase and its intramolecular synergy.** Shown are activities of artificial bi-catalytic cellulase (uppermost curve), its individual catalytic protein subunits (lowermost two curves), and a simple mixture of the two catalytic subunits (second curve from top). All cellulases were previously complexed with a CBM3a-bearing, single-cohesin scaffoldin binding adaptor, and all were loaded at the same (2.0 μmol/L) concentration, acting against Avicel substrate loaded at 5 g/L, for an enzyme: substrate ratio of 0.4 μmol per g cellulose. Assays were carried out anaerobically at 60°C in 20 mM acetate, pH 5.0, containing 10 mM CaCl_2_, 5.0 mM L-cysteine and 2 mM EDTA. In addition to the cellulases under study, each assay mixture included chromatographically purified *Aspergillus niger* β-glucosidase at a concentration of 0.005 mg/mL (or 1.0 mg/g of cellulose substrate). For key to icons, please see Figure 1 and Table 1.

Given the current limitations of minicellulosomes constructed using only cellulosomal enzymes with one catalytic module, an artificial multifunctional cellulase intended for incorporation into new minicellulosomes was constructed by fusing a truncated version of a processive endoglucanase, *C. thermocellum* CbhA (CBM4-Ig-GH9-X1_1_-X1_2_, tCbhA) and a classical endoglucanase, Cel8A (GH8-Doc) resulting in a new molecule with architecture of CBM4-Ig-GH9-X1_1_-X1_2_-GH8-Doc (Table 1 and Figure 1). In this artificial enzyme, the two consecutive X1 domains were considered special “spacer” or “linker” segments between two component peptide domains [27,28]. This large protein was found to be soluble and stable when overexpressed in *E. coli*. The activity of this multifunctional cellulase was measured on Avicel and compared with the activity of the truncated CbhA (tCbhA, in which the CBM3b module had been deleted) and Cel8A both individually and in a simple mixture (Figure 2). All of these constructs were complexed with a Coh-CBM3 partner to provide stronger binding to cellulose.

**Table 1:** Expressed proteins prepared in this study

**Protein**	**Module architecture**	**Gene source**	**Plasmid**	**Tag**
Modified Cel48S-T	CBM4-Ig-GH48-Doc^A^	*C. thermocellum*	pET28	C-terminal His-tag
Modified Cel48S-C	CBM4-Ig-GH48-Doc^B^	*C. thermocellum C. cellulolyticum*	pET28	C-terminal His-tag
Cel8A	GH8-Doc	*C. thermocellum*	pET28	C-terminal His-tag
Truncated CbhA	CBM4-Ig-GH9-X1_1_-X1_2_-Doc^C^	*C. thermocellum*	pET22	C-terminal His-tag
Bi-functional cellulase	CBM4-Ig-GH9-X1_1_-X1_2_-GH8-Doc^D^	*C. thermocellum*	pET22	C-terminal His-tag
Truncated CipA	Coh-CBM3a^E^	*C. thermocellum*	pET28	C-terminal His-tag
Chimeric scaffoldin	Coh-CBM3a^F^	*C. thermocellum C. cellulolyticum*	pET28	C-terminal His-tag
Chimeric scaffoldin	CBM3a-Coh-Coh^G^	*C. thermocellum C. cellulolyticum*	pET28	C-terminal His-tag

Note: GH, glycoside hydrolase family; CBM, carbohydrate binding module; Coh, cohesin; Doc, dockerin; Ig, immunoglobulin-like fold. ^A^The “CBM4-Ig” sequence segment is derived from *C. thermocellum* CbhA, and “GH48-Doc” from Cel48S. ^B^“CBM4-Ig” and –“GH48” are derived from *C. thermocellum* CbhA and Cel48S respectively, but here, “Doc” is from *C. cellulolyticum* Cel48F. ^C^Effectively the sequence of CbhA with the CBM3b excised. ^D^“CBM4-Ig-GH9-X1_1_-X1_2_” is the N-terminal portion of CbhA, and “GH8-Doc” is the sequence of *C. thermocellum* Cel8A. ^E^Excised internal segment of *C. thermocellum* CipA (Coh2 and CBM3a). ^F^ “Coh” is the Coh1 of *C. cellulolyticum* CipC and “CBM3a” is from *C. thermocellum* CipA. ^G^“CBM3a” is from *C. thermocellum* CipA; the first “Coh” is the Coh1 of *C. cellulolyticum* CipC, and the second “Coh” is the Coh4 of *C. thermocellum* CipA.

The uppermost two curves in Figure 2 compare the cellulolytic activity of this multicatalytic cellulase with the activity of an equimolar mixture of tCbhA and Cel8A. The activity of the multicatalytic construct is substantially greater than that of the simple enzyme mixture. The traditional approach to assigning a numerical value to the synergistic effect is to compare product release after the same (fixed) reaction time [29,30]. In this approach, the saccharification of 50.2% of the substrate in 70.3 h by the multifunctional construct, compared with conversion of only 32.3% of the substrate by the same molar loading of the individual components in a simple mixture, yields a synergism index of 1.56.

However, an alternative comparison that may be more meaningful is comparing the reaction times required for the two enzyme preparations to convert equal and significant fractions of the available substrate. In this case, the two enzyme systems are compared on the basis of time required to achieve the same extent of conversion of the substrate. This extent-of-conversion target line has been inserted in Figure 2, with the value of 32% conversion chosen to minimize inaccuracies arising from linear interpolation between data points by placing the intersection-points on each curve as close as possible to actual data points. The two drop-lines from the intersections of the uppermost two curves with this conversion-target line indicate that the linked, multicatalytic construct requires only 15.6 h to solubilize 32% of the substrate, whereas the simple mixture of the two catalytic domains requires 67 h. Because the reciprocal of the time required for an enzyme reaction to reach a given extent of completion is inversely proportional to the enzyme activity loaded [31,32], we can state that the multicatalytic construct has almost 4.3 times the activity of the equivalent mixture of its individual components.

### Construction of a targeted GH48-containing cellulosomal subunit

To complement the activity of the multifunctional cellulase described above when incorporated into minicellulosomes, we used a variant of the exoglucanase Cel48S from *C. thermocellum* to promote endo/exo synergism. Cel48S is considered to be one of the most important cellulases in the *C. thermocellum* cellulosomal system, on the basis of both *in vitro* chemical experiments [11,33] and the significant effect of its knockout *in vivo* on the cellulolytic activity of *C. thermocellum*[34]. Also, Cel48S has been described as playing a key role in the extremely high activity of native cellulosome on crystalline cellulose [11,33]. Heterologous expression of this specific protein at large scale in a soluble form has thus far proven to be extremely difficult. Although the recent addition of CBMs as purification tags has improved the ability to isolate Cel48S in soluble form, the production of the protein in *E. coli* is only possible in small quantities [35]. Thus, our sequence of objectives with regard to Cel48S was to make it more amenable to *E. coli* expression, obtain soluble recombinant protein, and to use this key protein in a minicellulosome. The two consecutive *N*-terminal modules of *C. thermocellum* CbhA (CBM4 and the Ig-like tandem repeat) were fused to the *N*-terminal peptide of Cel48S to generate a new cellulase, Cel48S-T (CBM4-Ig-GH48-Doc). This modified Cel48S construct can easily be overexpressed in *E. coli* and is partially soluble. The addition of BSA at 0.5% (w/v) is required for long-term storage of the recombinant protein in Tris buffer (20 mM Tris, pH 7.0, 100 mM NaCl, 2 mM CaCl_2_ and 0.02% sodium azide). Based on our successful expression of Cel48S-T and in order to incorporate this cellulase into a specific site in a minicellulosome (by means of a divalent scaffoldin to be described below), the dockerin in Cel48S-T was replaced by that of *C. cellulolyticum* Cel48F, creating a new gene product, Cel48S-C. The overexpression in *E. coli* of the Cel48S-C yielded a recombinant protein with solubility that was similar to that of Cel48S-T.

### Cellulase activity of modified Cel48S subunit and endo-exo synergism with multicatalytic subunit

**Figure 3:**
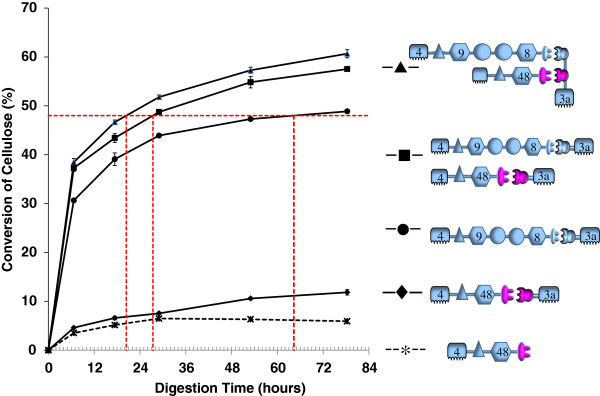
**Avicelase activity of the designer minicellulosome and its proximity synergy.** Activities of the minicellulosome (uppermost curve), its individual catalytic protein subunits (lowermost two curves), and a simple mixture of the two catalytic subunits (second curve from top) were analyzed. As in Figure 2, all catalytic protein subunits (with the exception of the lowermost curve, which is presented as a dashed line) were previously complexed with a CBM-bearing binding adaptor, either a single-cohesin scaffoldin or, in the case of the minicellulosome, a two-cohesin scaffoldin with each of the two cohesins specific for the dockerin module borne by one of the catalytic subunits. The lowermost (dashed) line represents Avicel conversion by a “bare-dockerin” version of the modified Cel48S subunit, i.e., one not provided with the *C. cellulolyticum*-cohesin/CBM3a binding adaptor matching its dockerin. Assay conditions as in Figure 2. For key to icons, please see Figure 1 and Table 1.

Figure 3 shows that sugar-release by Cel48S-C from Avicel is very low compared to the 48.9% converted by the multicatalytic cellulose in 78.3 h, in that Cel48S-C reached only 11.7% conversion of Avicel in the same length of time. Conversion by the simple mixture of artificial multifunctional cellulase and the modified Cel48S-C was 57.5%, yielding a conventional synergism factor of 0.95 for the two proteins. However, when we apply the more meaningful time-to-target approach, using a convenient target-level of 48% conversion, we see that the simple mixture of the multicatalytic enzyme and Cel48S-C releases 48% of the potential glucose in only 28 h, whereas the multifunctional enzyme alone requires 69.6 h to do the same. In other words, when we add an equal loading of the apparently much weaker enzyme to the loading of the “stronger” enzyme, thus increasing the total enzyme protein loading by a factor of 2.0, the activity of the mixture is 2.5 times the activity of the original loading of the “stronger” enzyme. This indicates a very significant cooperative, as opposed to merely additive, interaction between the two activities.

### Intermolecular and proximity synergy analysis using designer minicellulosomes

**Figure 4:**
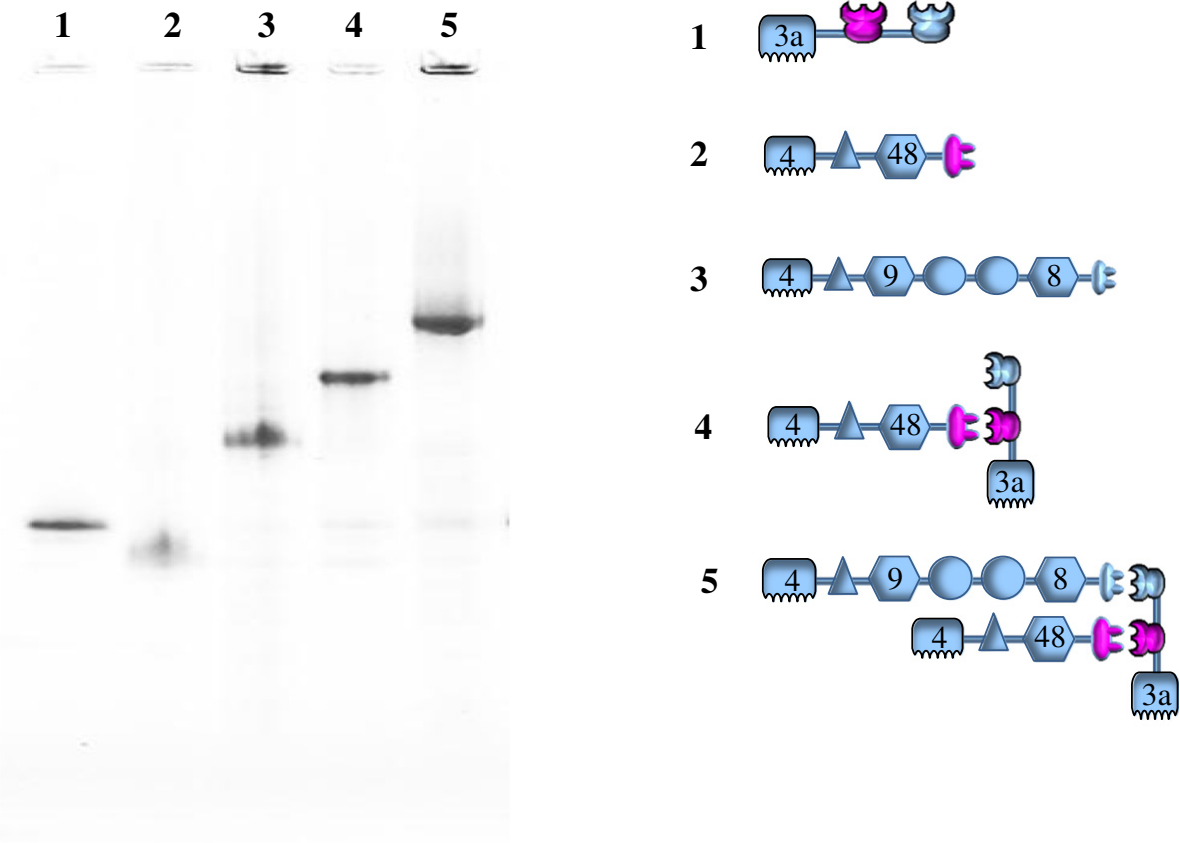
**Assembly of the minicellulosome and its verification by native PAGE.** Lane 1, chimeric scaffoldin (CBM3a-Coh-Coh); 2, modified Cel48S (CBM4-Ig-GH48-Doc); 3, artificial multicatalytic cellulase (CBM4-Ig-GH9-X1_1_-X1_2_-GH8-Doc); 4, complex formed by mixing chimeric scaffoldin and modified Cel48S; 5, completed minicellulosome formed by mixing artificial multicatalytic cellulase with complex shown in Lane 4. For key to icons, please see Figure 1 and Table 1.

In order to investigate the synergism of the multicatalytic and Cel48S-C cellulases in a minicellulosome, we constructed an empty chimeric scaffoldin including a CBM3a and two different cohesins (Figure 1 and Table 1), each specific to the dockerin on one of the cellulases. The proper assembly of the minicellulosome is demonstrated by native gel electrophoresis, shown in Figure 4. The modified Cel48S-C is first bound to one cohesin of the scaffoldin, resulting in a single protein band on native PAGE (Figure 4, Lane 4). Subsequently, the multicatalytic cellulase is added and allowed to bind to the other cohesin, again producing a single band with higher molecular weight, demonstrating that the desired minicellulosome has been constructed as designed (Figure 4, Lane 5). The cellulase activity of the minicellulosome was then assayed and the digestion curves are shown in Figure 3. The two uppermost curves are the central finding of our study; the connection of the multicatalytic enzyme to Cel48S-C through a two-cohesin scaffoldin produces a minicellulosome with activity greater than that of an equivalent simple mixture of the individual enzymes, each bound to a single-cohesin scaffoldin. In 78.3 h of digestion, the two-cohesin minicellulosome saccharifies 60.7% of the substrate, compared to 57.5% conversion by the mixture of separate, single-cohesin-equipped enzymes. These results yield a traditional synergism factor of 1.05, a small but statistically significant improvement given the relatively small errors in the data. The more meaningful time-to-target approach indicated that the minicellulosome converted 50% of the substrate in 25 h, which is to be compared with the 34 h time required by the mixture of the separate, single-cohesin-equipped enzymes. This time to target comparison yields a higher synergism factor of 1.36.

Consideration of the reported possible instability of mesophile-derived *C. cellulolyticum* cohesin domains (17) could quite justifiably raise concerns about the ability of the chimeric scaffoldin used in this study to survive the 60°C assay conditions used. At the time these experiments were designed, the *C. cellulolyticum* cohesin modules were the only alternative available for use in conjunction with *C. thermocellum* cohesins to achieve selective targeting of cellulosomal subunits to specific sites on the miniscaffoldin. Fortunately, it would appear from the activity-assay results that the *C. cellulolyticum* cohesin module (in combination with its matching dockerin) may retain atleast a substantial fraction of its function throughout the course of the 78-h assay.

The lowermost curve in Figure 3 (the only curve presented as a dashed line) tracks the individual activity against Avicel of a “bare-dockerin” version of the modified Cel48S subunit, i.e., the same catalytic polypeptide used for all of the other Cel48S-containing assays portrayed in Figure 3, but without the cohesin-CBM3a binding adaptor provided to the enzymes in the other assay-sets. This lowermost curve should be compared with the progress-curve immediately above, which tracks Avicel conversion by the same polypeptide when pre-complexed with the cohesin-CBM3a binding adaptor. These curves demonstrate that at 60°C the two versions of the modified Cel48S are similar in activity up to approximately 29 h and 6.5 - 7.5% conversion. Past that point, the enzyme that was never connected to the CBM3a through the *C. cellulolyticum* cohesin module appears dead in the water, perhaps unable to find anything else to hydrolyze. The modified Cel48S that was supplied with the *C. cellulolyticum*-cohesin/ CBM3a adaptor continues to churn out more soluble sugar, up to the termination of the assay at 78.3 h. It is not unreasonable to infer that the difference between the two curves is that the modified Cel48S that was supplied with the binding adaptor still has the binding adaptor in the latter part of the digestion, because the cohesin-dockerin interaction is still intact under the exact conditions of the assay.

Further support for this idea is provided by the next two curves up in Figure 3 (second and third from the top), representing digestions by the binding-adaptor-supplied multicatalytic construct alone (third curve from top), and in simple mixture with the binding-adaptor-equipped modified Cel48S (second curve from top). From 29 h onward, the Cel48S/multicatalytic mixture is seen not only to be maintaining its lead over the multicatalytic enzyme alone, but to be pulling away. It would seem unlikely that this would have happened if the Cel48S had reverted to the late-stage activity-level shown by the “bare-dockerin” version because the *C. cellulolyticum* cohesin had unraveled, taking away the added CBM3a. If it should be the case that some portion of the *C. cellulolyticum*-cohesin/dockerin pairs of the minicellulosome did disintegrate over the course of the assays, this would imply that the synergism is actually greater than the synergism observed.

## Discussion

In Nature, there are three major types of cellulase synergy: that resulting from complementary interaction of separate endo- and exo-cellulases, the intramolecular synergy of multifunctional cellulases and finally the longer-distance proximity-dependent synergy observed in cellulosomes[2,4,6]. Intramolecular synergy has been demonstrated earlier by some multicatalytic cellulases and hemicellulases both in native and artificial systems [5,13,14]. Our present results show that when two catalytic modules, namely GH9 and GH8, are connected by a particular linker peptide, the resulting new multifunctional cellulase displays high intramolecular synergy and also is the first reported artificial cellulosomal multifunctional cellulase demonstrating high activity on crystalline cellulose. This supports our conclusion that construction of new cellulosomal multifunctional cellulases is not only a promising approach to enhance activity of collections of catalytic modules, but also has the potential to further improve the activity of minicellulosomes by incorporation of these multicatalytic cellulases.

The fact that this relative increase in activity is smaller than that seen for the combination of GH9 and GH8 modules in a single construct to make the multifunctional peptide (Figure 2) may reflect the diminishing returns encountered when more active enzymes, having progressed further through the substrate, subsequently encounter steadily more recalcitrant material. An alternative explanation may also be the more significant increase in synergy that occurs in the first step of construction, creation of the multifunctional cellulase from individual catalytic modules, which being already localized, gain little additional benefit from being tethered together further.

In most recent related studies, the minicellulosomes reported contained a single catalytic module in each cellulosomal component, and therefore utilized only two types of synergy; the synergy of complementary activities and the long range proximity-dependent synergy. These constructs showed low conversions of crystalline substrates, although the low conversions reported in these studies can also be attributed to the relatively low protein loadings used [15-17]. The protein loading used here and the use of a multicatalytic cellulase enable the minicellulosome to achieve a high extent of conversion of crystalline cellulose never seen before for minicellulosomes.

Multicatalytic peptides offer important advantages in the construction of minicellulosomes. Consolidated bioprocessing (CBP), the idea of combining cellulase production, cellulose hydrolysis, and soluble sugar conversion to biofuels into one single step is one potential approach to reduce the cost of producing lignocellulosic biofuels [36-38]. In order to enhance the capability of cellulose degradation in non-cellulolytic CBP species (for example, yeast) minicellulosomes have been introduced and the modified strains have showed promising capability in degrading cellulose [18,20,21]. It is possible that our new concept of multicatalytic enzymes in minicellulosomes described above could help engineer better CBP organisms with a reduced number of cellulase genes and improved activity.

## Conclusion

Compared to other reported minicellulosomes that were assembled from cellulases containing only one catalytic module each, our new minicellulosomes display the distinct advantages of reduction in the number of cellulases required for the assembly of minicellulosomes and easier co-expression of minicellulosome genes in microbial hosts. These results show promise for use of minicellulosomes both as tools for exploring cooperative enzyme interactions in deconstruction of cellulosic materials and as eventual practical catalysts in biomass conversion processes.

## Materials and methods

### Cloning

Some of the genes, or gene-segments encoding individual domains, were synthesized by GenScript (http://www.genscript.com); others were cloned in our laboratory. Some cellulase genes were amplified from the genomic DNA of *C. thermocellum* (ATCC 27405); the primers used in this process are listed in Table 2. The genes encoding the cellulases, scaffoldins, dockerin-replaced cellulases, and multifunctional cellulases were constructed and cloned by standard cloning methods [39]. The architectures of all gene-products used in this study are listed in Table 1 and presented pictorially in Figure 1.

**Table 2:** Primers used for this study

**Primer**^**A**^	**Nucleotide sequence**^**B**^	**Gene cloning or construction**
F-V8-NdeI	TCCGTGCATATGTTAGAAGATAATTCTTCGACT	CbhA
R-V8D-XhoI	CTGTACCTCGAGATCCCGTGCCTGTTTTACAA	CbhA
F-NcoI-cels1	CTGCATCCATGGGTCCTACAAAGGCACCTAC	Cel48S
R-XhoI-cels1	ATCAGTTTTGCTCGAGGTTCTTGTACGGCAATGTAT	Cel48S
F-V7-NcoI	CTGTGTCCATGGCAGGTGTGCCTTTTAACACA	Cel8A
R-V7-XhoI	CCCATTCTCGAGATAAGGTAGGTGGGGTATGC	Cel8A
F-V7-XhoI	ACTGTGCTCGAGGCAGGTGTGCCTTTTAA	Cel8A

Note: ^A^Other genes listed Table 1 were synthesized by GenScript, and their primers are not listed. ^B^Residues underlined are the designated restriction enzyme cleavage sites.

### Gene overexpression and recombinant protein purification

All genes encoding the scaffoldins, cellulosomal cellulases and engineered multifunctional cellulases were overexpressed in the BL21(DE3) strain of *E. coli* (Stratagene, La Jolla, CA) in the presence of 0.3 mM IPTG at either 16 or 37°C. All recombinant proteins were purified by Nickel-NTA affinity chromatography (Table 1).

### Assembly of minicellulosome

Purified wild-type and engineered enzymes were mixed in equal molar amounts with an engineered scaffoldin to form minicellulosomes. Purified chimeric scaffoldin was first mixed with Cel48S-C (both stocks in 20 mM Tris, pH 7.0, 100 mM NaCl, 2 mM CaCl_2_ and 0.02% sodium azide) and allowed to incubate at room temperature for 30 mins to form the partial minicellulosome. The multifunctional cellulase was then added in the same way to form the complete minicellulosome. The formation of defined minicellulosome was verified analytically by native PAGE [15], using a gradient native gel (4-16%) purchased from Invitrogen (Carlsbad, CA). The verified minicellulosomes were then assayed for activity against microcrystalline cellulose (Avicel PH-101).

### Cellulase activity assay

Cellulase activity was measured under anaerobic conditions using microcrystalline cellulose (Avicel PH-101, Fluka; Sigma-Aldrich Corp., St. Louis, MO) as substrate. All enzymes were loaded at a standard molar concentration of 2.0 micromoles/L, working against a standard substrate (Avicel) loading of 5.0 mg/mL (for an enzyme: substrate ratio of 0.4 μmol enzyme per g cellulose). Assays were carried out at 60°C in 20 mM acetate, pH 5.0 containing 10 mM CaCl_2_, 5.0 mM L-cysteine and 2 mM EDTA to promote stability of the anaerobe-derived cellulases. In addition to the cellulases under study, each assay mixture included *Aspergillus niger* β-glucosidase (chromatographically-purified from the commercial mixture Novozym 188 (Novozymes North America, Franklinton, NC, USA.)) at a concentration of 0.005 mg/mL (or 1.0 mg/g of cellulose substrate), which loading is sufficient to maintain cellobiose concentrations below the levels at which cellobiose-inhibition of the enzymes is measurable.

Assays were carried out in triplicate, in initial digestion volumes of 1.0 mL in crimp-sealed 2.0-mL HPLC vials, with constant mixing by inversion at 10/min in a rotating incubator inside a glove box maintaining an atmosphere of 5% hydrogen, 95% nitrogen. At designated time-points during the digestions, representative 0.1-mL aliquots of liquid and solids were withdrawn for analysis, with the digestion vials being opened and then re-capped anaerobically inside the glove-box. The withdrawn aliquots of digestion mixture were diluted 18-fold with deionized water in sealed 2.0-mL HPLC vials, and then immersed for 10 min in a boiling water bath to terminate the enzyme reactions. The diluted digestion-mixture aliquots were then filtered (0.2-μm Acrodisc^R^) before quantification of released sugars by HPLC. HPLC sugar analyses were carried out on a Bio-Rad (Hercules, CA) HPX-87H column operated at 65°C with 0.01 N H_2_SO_4_ (0.6 mL/min) as mobile phase in an Agilent (Santa Clara, CA) 1100-series liquid chromatograph with refractive-index detection.

## Abbreviations

GH: Glycoside hydrolase family; CBM: Carbohydrate binding module; Coh: Cohesin; Doc: Dockerin; Ig: Immunoglobulin-like fold.

## Competing interests

The authors declare that they have no competing interests.

## Authors’ contributions

QX, JOB, RB, YJB, SYD, and MEH designed and coordinated the study, and revised the manuscript. QX conducted molecular biology work, and wrote the first draft. JOB conducted all enzyme assays. All authors read and approved the final manuscript.
